# Ultrasound-Enhanced Drug Delivery in Pediatric Neuro-Oncology: A New Therapeutic Strategy

**DOI:** 10.3390/pharmaceutics18050576

**Published:** 2026-05-07

**Authors:** Elora Weber, Christian A. Smith, Cynthia Hawkins, Uri Tabori, Peter B. Dirks, James T. Rutka

**Affiliations:** 1 Arthur and Sonia Labatt Brain Tumour Research Centre, The Hospital for Sick Children, 555 University Avenue, Toronto, ON M5G 1X8, Canada; siobhon-elora.weber@sickkids.ca (E.W.); christian.smith@sickkids.ca (C.A.S.); cynthia.hawkins@sickkids.ca (C.H.); uri.tabori@sickkids.ca (U.T.); peter.dirks@sickkids.ca (P.B.D.); 2 Department of Laboratory Medicine and Pathobiology, University of Toronto, Toronto, ON M5R 0A3, Canada; 3 Department of Pathology, The Hospital for Sick Children, Toronto, ON M5G 1X8, Canada; 4 Department of Haematology/Oncology, The Hospital for Sick Children, Toronto, ON M5G 1X8, Canada; 5 Department of Medical Biophysics, University of Toronto, Toronto, ON M5R 0A3, Canada; 6 Department of Neurosurgery, The Hospital for Sick Children, Toronto, ON M5G 1X8, Canada

**Keywords:** blood-brain barrier, magnetic resonance imaging, tumor immune microenvironment, glioma, medulloblastoma, focused ultrasound

## Abstract

Pediatric brain tumors are highly prevalent and remain one of the leading causes of cancer-related deaths in children. There are numerous different brain tumor types that are now well characterized by magnetic resonance imaging (MRI), patient clinical course, and neuropathological and molecular genetic alterations. One of the challenges with treating pediatric brain tumors with systemic chemotherapy is the inability of several chemotherapeutic agents to cross the blood–brain barrier (BBB), which serves as a protective mechanism for neuronal homeostasis. The BBB primarily comprises microvascular endothelial tight junctions. Controlling BBB permeability to allow for therapeutics to cross and combat brain tumors is now possible using MR-guided focused ultrasound (MRgFUS). In this approach, microbubbles are administered intravenously prior to MRgFUS BBB disruption at the targeted tumor site in the brain. In the presence of MRgFUS, the microbubbles in the brain capillaries oscillate and temporarily disrupt the BBB, enabling systemically administered chemotherapy drugs to cross at the targeted site. In this review, we provide evidence supporting the use of MRgFUS BBB disruption to treat brain tumors in animal models and in ongoing human clinical drug trials. We conclude with efforts to harness the potency of the immune system using MRgFUS against pediatric brain tumors.

## 1. Introduction: Therapeutic Barriers in Pediatric Brain Tumors

The clinical burden of pediatric brain tumors accounts for approximately 23% of new childhood cancer cases under the age of 15 in Canada alone, and they are the most common cause of cancer-related mortality in children under 14 years of age in the United States, at an incidence rate of approximately 2.70 and 3.03 per 100,000 in Canada and the United States, respectively [1,2]. Despite advances in the treatment of central nervous system (CNS) tumors, systemic therapies frequently fail due to the restrictive properties of the blood–brain barrier (BBB) and a heterogeneous tumor immune microenvironment (TME). The BBB is a selective protective mechanism that controls the delivery of molecules from the blood to the brain and maintains CNS homeostasis [3]. However, this protective mechanism also limits the delivery of systemically administered therapeutics to the brain. The blood–brain tumor barrier (BBTB) refers to the often more permeable or “leaky” BBB, caused by new blood vessels and malignant glioma tissue, located in the core of the tumor [4,5]. There are physical and metabolic barriers in the BBTB [5]. The tight junctions between the microvascular endothelial cells produce the physical barrier while the drug efflux transporters and endosomal sorting processes produce the metabolic barrier [4]. Additionally, the barrier is surrounded by astrocytes, oligodendrocytes and pericytes, all interconnected with neurons and microglia [4,5]. The microenvironment of the BBTB consists of cell chemokines, growth factors and proteolytic enzymes, all favoring tumor proliferation, invasion, adhesion, angiogenesis, and chemotherapy resistance [5]. Therefore, the structural and functional heterogeneity of the BBTB and the microenvironment are strong factors that determine immunotherapeutic function.

### Knowledge and Therapeutic Opportunies for Pediatric Brain Tumors

Advances in genomic and epigenomic profiling have recognized distinct molecular subgroups across pediatric brain tumors, including medulloblastoma, ependymoma, and glioma [6,7]. These tumors differ from each other in their molecular drivers and their BBB integrity, which influences their therapeutic response. Classifying and understanding these tumors based on their features is critical for designing rational delivery strategies for emerging immunotherapies. Magnetic resonance-guided focused ultrasound (MRgFUS) has emerged as a non-invasive approach capable of transiently opening the BBB while simultaneously modulating the TME. Notably, the clinical translation of MRgFUS in pediatrics is supported through the established safety profile of both focused ultrasound and intravenously administered microbubbles (MBs) [8]. MBs have been widely used as ultrasound contrast agents, evolving into therapeutic agents in the adult and pediatric population with well characterized pharmacokinetics and minimal reported adverse side effects [8]. Furthermore, clinical studies utilizing MRgFUS-mediated BBB disruption have shown that BBB opening is achieved through a controlled, transient and reversible approach, without significant tissue damage or neurotoxic side effects. While most research has focused on adult brain tumor models and patients for MRgFUS-mediated BBB disruption, this strategy presents a suitable method to target pediatric tumors, limiting the risk of toxicity to the developing brain [9]. This review synthesizes current knowledge on BBB/BBTB heterogeneity across the five major pediatric brain tumor types and discusses how MRgFUS-mediated BBB disruption can be leveraged to overcome structural and functional limitations, through enhancing immunotherapeutic delivery and potentially modifying the pediatric tumor immune microenvironment. We highlight key differences between pediatric-specific evidence and findings derived from adult and preclinical studies, with emphasis on further pediatric investigation. We conclude by discussing combination strategies with MRgFUS that can be used for specific tumors based on their TME composition and the need for individual patient-specific stratification.

## 2. The Blood–Brain Barrier, Blood–Brain Tumor Barrier and Tumor Immune Microenvironment in Pediatric Brain Tumors

In the presence of a tumor, the BBB architecture becomes disrupted due to abnormal vasculature, altered endothelial signaling, and elevated interstitial pressure [5]. The BBB may become compromised, leading to dysfunctional permeability and difficulty in managing immunotherapeutic treatment.

The TME represents a complex, immunosuppressive ecosystem, diversified with immune cell types, cancer cells, endothelial cells, adipocytes and neurons [10]. The myeloid composition varies extensively in the location of the tumor, tumor type and tumor subtype [10]. Notably, pediatric and adult brain tumors exhibit marked differences in TME composition and their baseline effector immune cell populations [11,12]. These differences may be influenced by age-dependent processes in the CNS influencing microglial populations, as microglia exhibit heightened neurodevelopmental activity, such as pruning neuronal networks and modulating astrocytes as the immune system is developing [12,13]. Researchers have begun to classify brain tumors based on their proteomic immune signatures. For example, low-grade gliomas and high-grade gliomas are characterized by the presence of macrophages, microglia, and dendritic cells; craniopharyngiomas are characterized by PD-1 and CTLA-4 expression; and ependymomas and medulloblastomas are characterized by lower immune infiltration [14].

Immunosuppression of the TME is caused by the release of immunosuppressive molecules such as IL-10, M2-like tumor-associated macrophages (TAMs), and the overexpression of programmed death ligand 1 (PD-L1), leading to a definitive TME structure that influences antitumor immune responses [15]. Collectively, the heterogeneity of tumors regarding their BBB integrity, myeloid polarization state, and baseline T-cell composition influences the rational selection of immunotherapeutic strategies [11,12]. We have summarized these defining features within the five common pediatric brain tumors and their subtypes including medulloblastoma, diffuse midline glioma, ependymoma, craniopharyngioma, and low-grade glioma.

### Tumor-Specific BBB/BBTB Heterogeneity and TME Baseline Composition

Medulloblastoma: This is the most common malignant childhood brain tumor, accounting for approximately 15% of all pediatric brain tumors [16,17] and includes the following four subgroups: Wingless-related integration site (WNT), Sonic Hedgehog (SHH), group 3, and group 4 medulloblastomas [16,17]. Within the four subtypes, SHH and group 4 medulloblastomas have an intact BBB, WNT medulloblastomas have a leaky BBB, and group 3 medulloblastomas have a mildly disrupted BBB [16,17]. The leaky BBB status of the WNT subtype permits immunotherapeutic systemic delivery into the tumor tissue, typically resulting in a survival benefit as opposed to the other three medulloblastoma subtypes, and has less of a requirement for MRgFUS-mediated BBB disruption [4].

Medulloblastoma is characterized by low levels of pro-inflammatory cytokines and T-cell infiltration, suggesting an immunosuppressive TME [18,19]. Within the SHH and WNT subtype, the TME is characterized by a predominance of TAMs, such as microglia and macrophages, whereas groups 3 and 4 have a higher expression of CD8+ T cells [12]. The SHH subgroup in particular exhibits more inflammatory cytokines than the other subgroups, whereas group 4 contains high levels of lymphocytes and neutrophil infiltration, with high expression of CD3+ T cells [19]. Overall, medulloblastomas express more M2-like macrophages than M1-like macrophages, few T-lymphocytes, and have low PD-L1 expression which leads to tumor proliferation and invasion [12,19,20,21].

Diffuse midline glioma (DMG): A subset of DMG is diffuse intrinsic pontine glioma (DIPG), which is the leading cause of pediatric brain tumor deaths, accounting for 10% of all pediatric high-grade gliomas and 80% of brainstem tumors [22,23]. DIPG has an intact BBB and a highly immunosuppressive TME, defined by a higher M2-like TAM to M1-like TAM expression ratio [17,20]. Its non-inflammatory TME is further characterized by low inflammatory marker and cytokine expression, reduced CD3+- and CD8+-infiltrating T cells, low mutational burden, and reduced antigen presentation [24,25,26,27].

Ependymoma: This is the third most common pediatric brain tumor, with four subtypes, two of which are in the posterior fossa and the other two in the supratentorial space [28,29,30]. Pediatric ependymomas have an intact BBB; however, within the supratentorial subtype, the BBB status is compromised [17,31]. Posterior fossa ependymomas have a low T-cell tumor infiltration and a low mutational burden, producing an immunosuppressive TME, and they are the most common ependymoma subtype to occur within pediatric patients [25,27,29,31]. Pediatric supratentorial ependymomas typically have a higher survival rate, as total gross surgical resection can be achieved, limiting the need for MRgFUS [32].

Craniopharyngioma: Pediatric adamantinomatous craniopharyngiomas (ACP) is the predominant form of craniopharyngioma in the pediatric population, and is defined by a compromised BBB, elevated inflammatory markers and immunomodulatory cytokines, such as IL-6 and IL-10, within the TME [17,33,34]. Craniopharyngiomas have a recurrence rate of 25%, with this rate being exacerbated in some ACP patients [35]. Interestingly, genomic profiling data showed that ACP tumors are stable throughout recurrence, with the activation of the MAPK pathway being the leading cause of recurrence [35]. Overall, research shows ACP tumors foster an immunosuppressive TME [35].

Low-grade glioma: This group of common pediatric brain tumors includes diffuse low-grade glioma, with an intact BBB, and pilocytic astrocytoma, featuring a compromised BBB [17,36]. According to one study, pilocytic astrocytoma has the highest degree of macrophage infiltration within the TME and a highly activated M1 phenotype, which increases inflammation, leading to increased responsiveness to treatment [20]. This tumor typically expresses a pro-inflammatory immune microenvironment, with the presence of T-lymphocytes and an M1 phenotype [12]. Pediatric low-grade glioma fosters a moderately immunosuppressive TME, with reduced PD-L1 expression, high CD8+ T-cell expression and CD163 macrophages [37].

## 3. Magnetic Resonance-Guided Focused Ultrasound: Mechanisms of BBB Disruption

**Figure 1:**
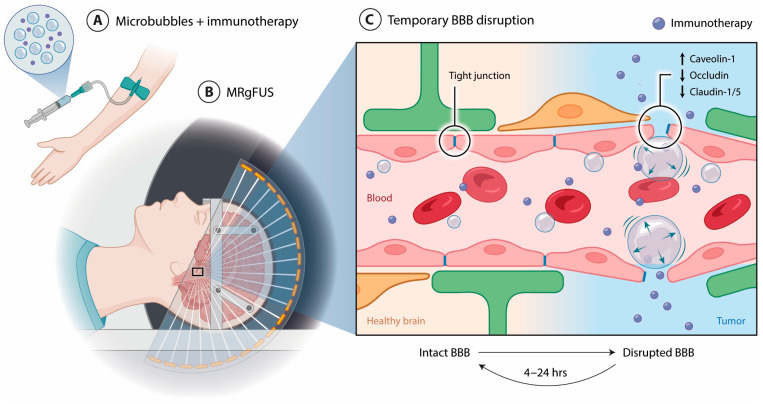
A focused ultrasound transducer is positioned over the patient’s head within the MRI patient table. (**A**) Microbubbles are administered intravenously along with selected immunotherapy. (**B**) The intracranial region is sonicated; MRI-guided focused ultrasound (MRgFUS) allows real-time image guidance and precise targeting of acoustic energy waves. (**C**) Upon sonication, the microbubbles interact with the ultrasonic field. At the microvascular level, microbubbles oscillate in the presence of ultrasonic waves, inducing mechanical stress on the endothelial cell wall, resulting in temporary and localized BBB disruption. This process is associated with the upregulation of caveolin-1, a key regulator in BBB permeability, and the downregulation of tight-junction proteins, including occludin, claudin-1 and -5. Collectively, this process increases BBB permeability, resolving within hours post sonication.

The biophysical mechanisms behind MRgFUS involve microbubble oscillation, mechanical stress on endothelial cells, tight-junction disassembly, and increased transcytosis [38]. MBs are injected intravenously and in the presence of low-intensity ultrasonic waves, oscillate, expanding and contracting, known as acoustic cavitation, to cause a temporary disruption of the BBB [4,39,40,41]. The absorbed ultrasound energy is transferred to the microbubbles, causing oscillation, which then achieves minimal observable normal-tissue disruption [40]. The stress exerted on the endothelial cell wall, leading to a deformation of the cells for a temporary opening of the BBB, is caused from acoustic pressures sufficient to induce stable MBs cavitation [40,42]. Other molecular mechanisms behind temporary BBB disruption include the upregulation of caveolin-1, which leads to BBB permeability 1 h post sonication. In addition, tight-junction proteins such as occludin, claudin-1 and -5 are downregulated [40]. These mechanistic features of MRgFUS and intravenous MBs are non-invasive techniques, transmitting acoustic energy waves through the skull using ultrasonic transducers to target precise locations within the brain [41]. This opening of the BBB is temporary and allows for the targeted delivery of therapeutics to the tumor region (Figure 1) [4,41].

The safety and reversibility of BBB opening have been studied extensively, enabling the implementation of MRgFUS devices in clinical settings. The transient opening of the BBB lasts for approximately 6 h, after which time the BBB restabilizes, remaining fully impenetrable thereafter [40]. Additionally, in animal models and adult human patients, there have been no reports of behavioral alterations, or impaired cognition or motor skills post BBB opening [40]. The entry of neurotoxins via the BBB has not been shown to exert adverse reactions, due to the activation of glial cells that can clear the components within the timeframe the BBB remains open [40]. MRgFUS overall has been demonstrated to be a safe and reliable method for delivery of therapeutic reagents to CNS tumors [39,40,41].

### MRgFUS BBB Disruption in Preclinical Animal Models

Preclinical studies demonstrate that MRgFUS-mediated BBB disruption significantly enhances intratumoral chemotherapy delivery and accumulation, translating to improved therapeutic efficacy and minimal toxic side effects in brain tumor models [43]. Doxorubicin, a chemotherapeutic, is non-BBB-penetrant. Delivering this drug via MRgFUS allows for the temporary disruption of the BBB and treatment of the targeted brain region [41,43]. A study using a DIPG mouse model that received a systemic injection of doxorubicin and microbubbles via MRgFUS led to an increased intratumoral concentration of the drug [41]. Cisplatin is another chemotherapeutic that is highly effective against several cancer types; however, nephrotoxicity and neurotoxicity are reported side effects in adult patients [44]. The use of MRgFUS with cisplatin has reduced some of its toxic side effects by controlling the delivery of the drug to targeted brain regions in mouse glioma models [44]. In a mouse model of glioblastoma (GBM), MRgFUS-mediated BBB opening with a systemic injection of etoposide increases the intratumoral concentration of this chemotherapeutic, which resulted in a concentration that was 8 times higher in brain tumor tissue than it would be if not treated with the focused ultrasound [45].

Herceptin is a humanized monoclonal antibody and has a molecular size of approximately 150 kDa, hindering its ability to cross the BBB [46]. MRgFUS-mediated transport of Herceptin across the BBB occurred in a pivotal study demonstrating enhanced therapeutic delivery [46]. Notably, patients with brain cancer metastases from breast cancer have also benefitted from MRgFUS-induced BBB disruption, as demonstrated from a HER2/neu-positive breast cancer brain metastases brain study, in which patients received trastuzumab, a therapeutic agent that is effective against extracranial metastases, but has a large molecular weight otherwise precluding delivery [47].

The safety and efficacy of repeated MRgFUS-mediated BBB openings have also been evaluated in preclinical studies, as treating brain cancer tumors may require repeated MRgFUS sessions. Repeated BBB disruption was evaluated in rhesus macaques to determine the safety and efficacy of the procedure [48]. This study showed that repeated BBB disruption via MRgFUS produced no behavioral deficits, tissue damage, or visual functional deficits [48]. In a rat glioma model, it was demonstrated that the low-frequency ultrasound system reliably opened the BBB without any vascular injuries using three sessions per week [49]. Preliminary work on female Sprague Dawley rats demonstrated the safety and efficacy of opening the BBB using MRgFUS and MBs [50]. In this study, the rotarod apparatus and forelimb grip strength tests were assessed in the rats to evaluate their motor function post treatment, along with a histological assessment of the brainstem to analyze tissue damage post BBB opening [50]. As a prelude to treating DIPG in the brainstem, these researchers showed the efficacy of MRgFUS + MBs + doxorubicin treatment [50]. They provided evidence that the most effective method of concentrating doxorubicin in the brainstem was using the combination of MRgFUS + MBs + doxorubicin when compared to MBs alone, MRgFUS alone, and MRgFUS+ MBs with no therapeutic agent [50].

Collectively, these studies establish a method to overcome heterogeneous BBB permeability, increase intratumoral chemotherapy concentrations, deliver large molecular agents, and in several models, reduce tumor burden and increase survival benefit. While the data on pediatric brain tumor models remain limited, the consistency of MRgFUS-mediated delivery of therapeutic agents across brain tumor models provides a strong translational rational for evaluating this methodology in the pediatric brain tumor population.

## 4. Immunomodulatory Effects of MRgFUS

### 4.1. The Function of the Tumor Microenvironment

MRgFUS not only enhances drug delivery but also can alter the TME through mechanical and inflammatory signaling pathways. Coined by Reardon and Antonio, the TME can be described as an immunologic desert, which can evade immunity [51]. The function of the TME is to protect the tumor cells and support their growth against the immune system, promoting metastasis, angiogenesis, acidic pH, and contributing to immunotherapy resistance [11]. This environment is sensitive and can change following MRgFUS treatment, promoting the transition from this immunologic desert, also termed an immunologically “cold” tumor, to a more inflammatory or “hot” tumor [52]. This shift induces cellular responses such as increased TAMs and microglia and enables the probability of the microenvironment responding to immune checkpoint inhibitors (ICIs) [52]. The combination of MRgFUS and ICIs, or MRgFUS + Chimeric Antigen Receptor (CAR) T cells, are strategies used in specific tumor cases and have been shown to exert varying biochemical and mechanical effects that enable the transition from a cold to hot immune environment [11,26].

There is currently limited research examining the immunomodulatory effects from MRgFUS-induced BBB opening in pediatric brain tumors compared with adult brain tumors. Focused ultrasound technologies were initially developed for treatment of adult populations. Clinical use in pediatric patients received regulatory approval in 2020 [53]. As of 2025, only six studies have reported the use of transcranial focused ultrasound in pediatric patients [54], highlighting the early stage of clinical investigation in the pediatric population. Importantly, pediatric brain tumors differ biologically from adult brain tumors of the same histological subtype, including differences in the immune microenvironment. Pediatric brain tumors often exhibit an immunologically cold TME, characterized by a lack of immune cell infiltration [55,56,57,58,59]. Accordingly, the immunomodulatory effects induced by MRgFUS may differ from the observations seen in adult tumors and preclinical models.

Poor T-cell infiltration and weak baseline immune activation, often contributing to immunotherapeutic resistance to ICIs, define immunologically cold tumors. Therapeutic strategies are needed to promote immune cell recruitment and activation, converting these tumors into a pro-inflammatory “hot state” characterized by high T-cell infiltration, high interferon-γ signaling, strong antitumor immune response, and improved responsiveness to immunotherapeutic agents [57,58,60,61,62].

### 4.2. Converting Immunologically Cold Tumors to Immunologically Hot Tumors: Biochemical and Mechanical Modulation of the TME

MRgFUS-mediated BBB opening induces cellular and tissue stress, which can lead to inflammation and increased microglia and macrophage expression [63]. An initial localized sterile inflammatory response (SIR) from the mechanical force of MRgFUS-mediated BBB opening induces cytokine release, increases expression of localized adhesion molecules, enhances leukocyte recruitment, and increases endothelial activation [43,52,60,63]. An SIR can prime the TME for immune reactions and increased antigen exposure, supporting a shift to a more inflammatory TME [52,63]. Preclinical research has demonstrated this SIR activation in a glioblastoma animal model, increasing the expression of pro-inflammatory molecules such as astrocytes and microglia [52]. Changes to the interstitial fluid pressure within the tumor are also observed, improving tissue perfusion and immune cell motility [64]. The classification of myeloid cells and the baseline intrinsic composition of the TME can illustrate the response to MRgFUS-BBB opening [20,55,60,63]. Dendritic cells may also play a role in these responses, as the recruitment of these cells increases antigen presentation and can prime T cells to respond to tumor cells [20]. MRgFUS can release damage-associated molecular patterns (DAMPs), stimulating dendritic cell recruitment and elevated responses in IL-1, IL-18, and TNFα [61]. MRgFUS promotes antigen presentation through the increased concentration of interferon-γ, a decrease in IL-10, and the preservation of IL-4, TGF-β1, and TGF-β2. Preclinical models have also demonstrated increased expression of pro-inflammatory cytokines, and enhanced antigen presentation, shown through the maturation of dendritic cells [65].

Preclinical evidence in rat glioma models shows some support for MRgFUS immunomodulation. Interluekin-12 was delivered systemically (IL-12) in combination with MRgFUS to trigger an immune response against cancer cells [66]. MRgFUS surprisingly did not influence the T-lymphocyte population; however, increases in CD3+ and CD8+ occurred post MRgFUS exposure alone [66]. The combination of MRgFUS and IL-12 delivery produced the most significant increase in CD3+, CD8+ and regulatory T cells within the tumor region [66]. Interestingly, MRgFUS can be used for localized transcranial hyperthermia, a method used in a rodent glioblastoma model that demonstrated enhanced concentration and drug delivery in solid tumors [67]. This thermal stress reduces the interstitial fluid pressure in extracranial tumors, refines nanoparticle accumulation, and changes vessel permeability, altering the TME [67].

Therefore, MRgFUS can act as an effective modulator of the TME. Through both thermal and mechanical effects, MRgFUS can stimulate immune-related processes including the release of tumor antigens and inflammatory mediators [11,23]. These effects can trigger downstream biochemical signaling that influences TAMs and microglia, key regulators of the TME [11,23]. However, relatively few studies have investigated the intrinsic differences in the TME across tumor types and how it influences immunological responsiveness to MRgFUS [11].

### 4.3. Immune Checkpoint Inhibitors and MRgFUS Modulate the TME

The combination of immune checkpoint inhibitors (ICIs) and MRgFUS is a promising strategy to activate the immune system, enabling the elimination of tumor cells. The responsiveness of ICIs depends on the presence of pre-existing T cells within the TME, in which immunologically cold tumors such as medulloblastomas have a low baseline T-cell infiltration [12]. Under physiological conditions, checkpoint pathways such as PD-1/PD-L1 regulate CD8+ T-cell-mediated immune responses and prevent autoimmunity [68]. This pathway is exploited by some tumors. To avoid immune-mediated clearance, the tumors will upregulate PD-1,leading to dysfunctional or “exhausted” T cells [68]. PD-1/PD-L1 ICIs is the most often used cancer immunotherapy and has demonstrated effectiveness since initial studies in 2014 [68]. The purpose of MRgFUS use is to achieve uniform ICI distribution within the restricted area of the tumor tissue [68,69]. In a rat glioma model, MRgFUS mediated the delivery of anti-PD-1, which resulted in the promotion of CD4+ T and CD8+ T cells [69]. Anti-PD1 was also delivered via MRgFUS in the GL261 mouse model of glioblastoma, with similar results, increasing pro-inflammatory molecules in the tumor region [64]. Research has also suggested closed-loop MRgFUS improves the penetration of ICIs in GL261 tumors and modifies the tumor immune microenvironment by enabling the control of MB oscillation through acoustic emission feedback [64].

These findings support the notion that MRgFUS functions as a localized immune adjuvant treatment for brain tumors, exerting positive effects on immune cell composition and converting the TME into a more permissive microenvironment to respond to ICIs [23,62]. It is important to note that a proportion of patients receiving ICI therapy experienced immune-related adverse events, with reports suggesting that up to one-third of patients may experience toxic side effects [26]. MRgFUS may help mitigate these risks by enhancing localized delivery of ICIs to brain tumors while limiting systemic exposure. MRgFUS may reduce off-target immune activation in surrounding tissues as it enables spatially targeted therapeutic delivery.

### 4.4. Chimeric Antigen Receptor (CAR) T-Cell Therapy and MRgFUS Modulate the TME

**Figure 2:**
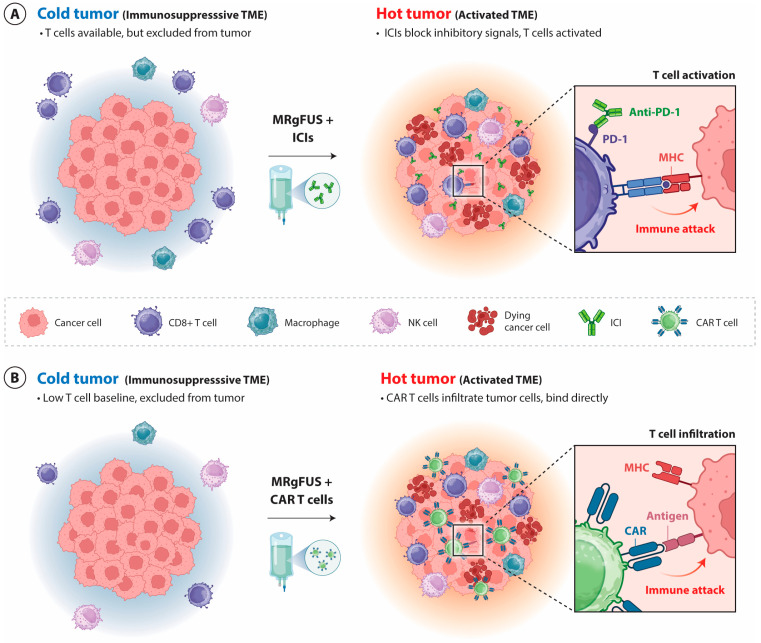
MRgFUS BBB modulates the TME and promotes immune priming through increased antigen release and stimulates immune-related processes. (**A**) MRgFUS in combination with ICIs mainly rely on antigen presentation (e.g., MHC system), and act on pre-existing T cells within the TME by blocking inhibitory signaling pathways such as PD-1/PD-L1, leading to the restoration of endogenous antitumor immune responses. (**B**) In contrast, when combined with CAR T-cell therapy, engineered T cells directly recognize tumor-associated antigens independent of MHC presentation, enabling direct cytotoxic activity even in tumors with low T-cell baseline infiltration. Despite these distinct mechanisms, both strategies promote immune activation within the TME, characterized by increased CD8+ cytotoxic T-cell infiltration, enhanced pro-inflammatory cytokine signaling, including IFN-γ and TNF-α from NK cells, and a shift towards immunologically inflamed TME.

Perhaps the newest immunomodulatory technique is CAR T-cell therapy. CAR T cells are genetically engineered T cells which respond to tumor antigens [70]. This novel technique allows each tumor and their genetic subtype to be targeted based on their antigenic expression [70]. This technique was developed in response to some cancers that can be unresponsive to ICI immunotherapies due to their limited ICI expression within the TME, particularly in pediatric DMG [26]. The efficacy of CAR T cells also appears to modulate TAM activity, successively modulating the TME [71]. There seems to be a required balance between pro-inflammatory and anti-inflammatory macrophages to prevent the failure of CAR T cells and their exhaustion [71]. The mechanism by which ICIs promote antitumor effects is by enhancing the current antitumor immune activity within the TME, whereas CAR T cells are engineered to respond to a specific tumor antigen, without the need for an immune checkpoint pathway inhibition, and can function independently from major histocompatibility complex (MHC) expression on tumor cells, thus directly affecting the tumor [72,73]. Figure 2 depicts the conversion of immunologically cold tumors into hot tumors following MRgFUS-mediated BBB disruption combined with either ICI therapy or CAR T-cell therapy.

Although CAR T cells are activated ex vivo and recognize tumor antigens independently, they can become exhausted or suppressed from myeloid populations within the TME [26]. There are some studies that suggest combining CAR T cells and ICIs will provide antitumor effects, as this can lead to reduced T-cell exhaustion [26]. Cytokine-armored CAR T cells can be engineered to secrete IL-12 and IL-18 to enhance antitumor immunity by promoting a pro-inflammatory microenvironment. These cytokines synergistically stimulate pro-inflammatory immune cells, such as T cells, NK cells and macrophages to produce IFN-γ, TNF-α, inhibit T-cell-mediated suppression, and promote the polarization of TAMs toward an M1-like pro-inflammatory state, amplifying the antitumor responses [74,75]. Such IL-12/IL-18-armored CAR T strategies may be particularly advantageous in immunologically cold tumors such as diffuse midline glioma and medulloblastoma, where endogenous immune activation is limited.

Immune effector cell-associated neurotoxicity syndrome (ICANS) is a recognized complication of CAR T-cell therapy in pediatric patients [76,77]. The developing brain in pediatric patients normally expresses higher levels of pro-inflammatory cytokines, which leads to neuroinflammation and can impact the severity of CAR T-cell-mediated ICANS [77]. Several limitations of CAR T-cell therapy, including severe toxicities, suppressive TME interactions, restricted trafficking to the tumor site, and antigen escape, can limit therapeutic efficacy [76,77,78,79]. MRgFUS has the potential to modulate these restrictions by enhancing delivery and altering the TME. Importantly, CAR T-cell therapy can function in TMEs with low endogenous T-cell expression, whereas ICIs typically rely on a pre-existing inflamed TME to achieve therapeutic efficacy. In addition, the tumor-associated extracellular matrix (ECM) represents a physical barrier that can restrict CAR T-cell infiltration and activity in solid tumors [80]. Heat-shock proteins, such as HSP47 and HSP90, contribute to ECM formation, in which MRgFUS has been demonstrated to mediate the heat-shock response, potentially influencing ECM structure to enhance CAR T-cell infiltration [80,81].

Collectively, incorporating specific tumor vasculature and immune cell populations into therapeutic stratification represents a critical determinant of immunotherapy success in pediatric neuro-oncology.

### 4.5. Case-Specific Approaches to Common Pediatric Brain Tumors

Pediatric patients present unique challenges compared with adults due to several physiological differences, which include an increased risk of perioperative hypothermia from their low weight-to-surface-area ratio, thinner skull and cap structures, and reduced subcutaneous adipose tissue deposits [82]. Beyond these physiological considerations, important immunological differences also exist between pediatric and adult brain tumors. Recently, research has outlined T-cell composition in common pediatric brain tumors, suggesting that immunotherapies that rely on the activation of pre-existing T cells in the TME, such as MRgFUS + ICIs therapy, may be less effective in the pediatric population as opposed to adults [31,60,83].

Immunotherapeutic strategies for medulloblastoma tumors are suggested through research showing that PD-1, PD-L1, and CTLA-4 antibodies have had effective results in the clinical pediatric population, except within the SHH subgroup, which possesses the highest PD-L1 expression of the four subtypes [83]. CAR T-cell therapy may represent an alternative strategy, as it can bypass the major histocompatibility complex (MHC) dependency of antigen presentation, directly target the tumor cells and enhance cytotoxic lymphocyte expression [13,19,21,79].

DMGs, particularly pediatric DMGs, often possess a cold TME, due to their lower levels of inflammatory marker expression and reduced CD3+-infiltrating T-lymphocytes [24]. There have been preclinical and clinical studies that show little to no support for the efficacy of ICI therapy, including MRgFUS-enhanced delivery [26]. Contributing to the reduced efficacy is the lack of immune checkpoint proteins and increased immunosuppressive markers via microglia promotion [26]. Interestingly, a study that used B7-H3 CAR T cells found immunomodulatory effects within the TME, such that there was an infiltration of tumor myeloid cells from the therapy, which reduces immunotherapy resistance [84].

Pediatric ependymoma RELA tumors demonstrate significantly higher PD-L1 expression compared to other ependymoma subtypes, suggesting ICI over CAR T-cell therapy [30]. However, one study showed that B7-H3-targeted CAR T cells were effective against treating both RELA and posterior fossa pediatric ependymomas, suggesting a personalized approach [83]. In ACP, recent studies support an MRgFUS + ICI approach, as the tumor expresses PD-L1, PD1, and high levels of inflammatory markers [33,34]. Additionally, a clinical trial using Tocilizumab, an IL6 inhibitor, has shown enhanced benefit within ACP patients [35]. Finally, low-grade gliomas have lower CD8+ T-cell and T-cell trafficking levels and reduced PD-L1 expression, suggesting MRgFUS + CAR T cells may be the appropriate strategy [37].

Table 1 summarizes the suggested case-specific approaches to common pediatric brain tumors based on their BBB and TME characteristics and past research.

As outlined in Table 1, the suggested strategies are highly dependent on tumor-specific characteristics such as immunological characteristics, which vary greatly between tumor types. This reinforces the need for personalized treatment approaches.

## 5. Clinical Translation: Ongoing Trials and Pediatric Interpretation

Safety and feasibility of MRgFUS BBB opening in patients were reported in a preliminary study recruiting a small sample size of five glioma patients [39]. This study served as the foundation that the BBB can be repeatedly and safely opened using MRgFUS, enabling targeted, transient disruption for the delivery of systemically injected chemotherapy, with no reports of dose-limiting toxicity [39]. Meng et al. were the first to report clinical evidence of targeted monoclonal antibody delivery across the BBB via MRgFUS to treat human patients with epidermal growth factor receptor 2- positive breast cancer brain metastases [85]. Importantly, these researchers showed MRgFUS increases the intratumoral concentration of trastuzumab, a chemotherapeutic, which can be applied to deep brain regions, such as the brain stem, cranial nerve nuclei, and cerebellum, offering a non-invasive adjunct approach for tumors located in surgically challenging regions. Additionally, BBB disruption was well tolerated in patients with no adverse side effects [85]. These results may serve as evidence that brain tumors that cannot be surgically resected, or do not respond to standard cancer treatments due to their location in the brain, may still benefit from MRgFUS BBB disruption to deliver therapeutic agents.

Current clinical trials investigating MRgFUS are exploring its potential to enhance the delivery of chemotherapeutics, such as temozolomide and carboplatin, in glioblastoma patients [86]. MRgFUS-mediated BBB disruption is also being explored to facilitate the transport of plasma cell-free DNA from GBM tumors into the bloodstream, enabling the use of liquid biopsy for diagnostics and treatment monitoring [87]. Liquid biopsy is a diagnostic approach used to analyze the pathology of tumors, such as the cerebral spinal fluid, blood, circulating tumor DNA and cells [88]. It serves as a real-time monitor of the tumor molecular environment, its response to therapeutic treatment, and its progression [88]. A recent study reported that transient MRgFUS-mediated BBB opening can increase the sensitivity of liquid biopsy by enriching the signal of circulating brain-derived biomarkers [88]. Patients with grade IV glioblastoma were recruited to evaluate the application of MRgFUS-based liquid biopsy, by delivering therapeutics and collecting blood samples at the same time to evaluate biological changes in the TME, suggesting that tumors can be treated individually according to their respective histological markers [88].

## 6. Future Directions

It will be interesting to see if MRgFUS-mediated BBB opening can be used for a growing number of pediatric brain tumors, or whether its application should be reserved for select tumor subtypes and patient-specific characteristics. Certain tumors such as DMG (DIPG), and the SHH subtype of medulloblastoma often retain an intact BBB, limiting conventional chemotherapeutic approaches. MRgFUS is required to enhance localized drug delivery and improve intratumoral distribution. MRgFUS should not be viewed as a universal drug delivery platform, but as a modulatory tool whose application is tailored to tumor biology, vascular characteristics, and therapeutic goals.

In parallel, emerging biomaterial strategies, including antioxidant and immunomodulatory hydrogels, have demonstrated an additional non-toxic approach to modulate pro-inflammatory signaling and macrophage polarization [89]. While this has not been explored in the pediatric population, this may become a complementary approach to MRgFUS-mediated immunotherapeutic delivery, highlighting the opportunity for integrating microenvironment-targeted therapies to treat pediatric CNS tumors [89].

Increasingly, using immune-oncology gene expression assays, recent research has elucidated significant heterogeneity within tumors of the same classification, particularly the composition of the TME [7]. Specifically, tumors that are recorded to possess an immunologically cold TME, for example DMG, were found to have extensive T-cell infiltrates and a strong PD-L1 positivity, suggesting that ICIs are a suitable option to treat certain patients with DMG [7]. This review highlights both preclinical and emerging clinical evidence supporting combination approaches involving MRgFUS with ICIs or CAR T-cell strategies. The selection of these approaches may depend on individual, rather than universal, tumor characteristics, including BBB integrity and the immunogenic profile of the TME.

## Figures and Tables

**Table 1:** Proposed immunotherapy strategies according to pediatric tumor type and their characteristics.

Tumor Type	BBB Status	TME Characteristics	Baseline T Cells	Suggested Strategy
DMG	■ Intact	■ Immunosuppressive	■ Very low	MRgFUS + CAR T
Medulloblastoma SHH	■ Intact	■ Moderate	■ Low	MRgFUS + CAR T
WNT	■ Leaky	■ More inflammatory	■ Moderate	MRgFUS + ICI
Group 3	■ Mild disruption	■ Immunosuppressive	■ Low	MRgFUS + CAR T
Group 4	■ Intact	■ Inflammatory	■ High	MRgFUS + CAR T or ICI
Ependymoma	■ Relatively intact	■ Moderate	■ Low	MRgFUS + CAR T
Supratentorial subtype	■ Compromised	■ Moderate	■ Low	Surgical resection
Craniopharyngioma (ACP type)	■ Compromised	■ Inflammatory	■ Moderate	MRgFUS + ICI
LGG Pilocytic Astrocytoma	■ Leaky	■ Inflammatory	■ Variable	MRgFUS + CAR T or ICI
Diffuse LGG	■ Intact	■ Moderate	■Variable	MRgFUS + CAR T or ICI

Therapeutic resistance: High ■ ■ ■ ■ ■ Low. Citations: DMG [19,22,26,27,28,29]; Medulloblastoma [4,14,18,19,20,21,22,23]; Ependymoma [19,27,29,31,33,34]; Craniopharyngioma [19,35,36,37]; LGG [14,19,22,38,39].

## Data Availability

No new data were created or analyzed in this study.

## Abbreviations

The following abbreviations are used in this manuscript:

ACP	Adamantinomatous Craniopharyngiomas
BBB	Blood–Brain Barrier
BBTB	Blood–Brain Tumor Barrier
CAR	Chimeric Antigen Receptor
CNS	Central Nervous System
DAMPs	Damage-Associated Molecular patterns
DIPG	Diffuse Intrinsic Pontine Glioma
DMG	Diffuse Midline Glioma
GBM	Glioblastoma
ICI	Immune Checkpoint Inhibitor
MRgFUS	Magnetic Resonance Imaging-Guided Focused Ultrasound
MHC	Major Histocompatibility Complex
MBs	Microbubbles
NK	Natural Killer
PD-L1	Programmed Death Ligand 1
SHH	Sonic Hedgehog
SIR	Sterile Inflammatory Immune Response
TAMs	Tumor-Associated Macrophages
TME	Tumor Immune Microenvironment
WNT	Wingless-related Integration Site
